# Resting-state EEG associated with clinical measures to predict upper limb motor recovery of subacute stroke

**DOI:** 10.3389/fneur.2025.1577393

**Published:** 2025-08-22

**Authors:** Ling Ding, Xiang Tian, Huiming Ren, Zihang Chen, Xiaokang Shu, Shugeng Chen, Jie Jia

**Affiliations:** ^1^The First Affiliated Hospital of Fujian Medical University, Fuzhou, Fujian, China; ^2^Shanghai Fifth People's Hospital, Shanghai, China; ^3^Department of Rehabilitation Medicine, Huashan Hospital, Fudan University, Shanghai, China; ^4^School of Mechanical Engineering, Shanghai Jiao Tong University, Shanghai, China; ^5^National Clinical Research Center for Aging and Medicine, Huashan Hospital, Fudan University, Shanghai, China; ^6^National Center for Neurological Disorders, Shanghai, China

**Keywords:** resting EEG, stroke, motor recovery, upper limb, prediction

## Abstract

**Background:**

After stroke, upper limb dysfunction seriously affects patients’ quality of life. The uncertain prognosis of patients poses a challenge for therapists in developing personalized rehabilitation programs. Electroencephalograph (EEG) power spectrum changes during rehabilitation training may have a predictive effect on the improvement of upper limb movement. Therefore, it is of great clinical significance to explore the EEG power spectrum related to the recovery of upper limb function after stroke.

**Method:**

This study included 113 subacute stroke survivors who were treated with routine rehabilitation for 2 weeks. At week 0 (T0) and week 2 (T2), behavioral scales including Fugl-Meyer Assessment of Upper Limb (FMA-UL), action research arm test (ARAT), modified Barthel index (MBI), and National Institutes of Health Stroke Scale (NIHSS) was assessed to compare correlations and observe the relationship between behavioral indicators and function under conventional rehabilitation. Twenty-six of the 113 patients were selected to undergo resting state EEG detection at week 0 (T0), week 1 (T1) and week 2 (T2), respectively. Power spectrum (PSD) and BSI values were calculated by EEG spectral analysis. The relationships between beta PSD and the clinical scales, between BSI and the clinical scales were examined by correlation and regression analysis.

**Results:**

Behavioral scales at T0 and T2 were positively correlated in both cohorts (*N* = 113, *N* = 26). Beta PSD correlated with FMA-UL (T1: *r* = 0.469, *p* = 0.016*; T2: *r* = 0.391, *p* = 0.048*) and ARAT (T0: *r* = 0.412, *p* = 0.037*; T1: *r* = 0.453, *p* = 0.021*; T2: *r* = 0.487, *p* = 0.012*). Beta BSI negatively correlated with Brunnstrom-UL (T2: *r* = −0.498, *p* = 0.01*), FMA-UL (T1: *r* = −0.441, *p* = 0.036*; T2: *r* = −0.507, *p* = 0.008*), and MBI (T2: *r* = −0.457, *p* = 0.019*). PSD-T0 predicted FMA-Hand (*β* = 0.997, *p* = 0.014*); PSD-T1 predicted ARAT (*β* = 1.945, *p* = 0.014*). BSI-T1 predicted Brunnstrom-Hand (*β* = −401.7, *p* = 0.049*) and FMA-UL (*β* = −194.4, *p* = 0.041*), demonstrating beta EEG’s prognostic value.

**Conclusion:**

Resting state EEG indicators, including beta PSD and BSI, may serve as prognostic biomarkers for upper limb motor function recovery of stroke survivors, providing valuable reference for further clinical decision-making.

## 1 Introduction

Stroke is one of the leading causes of disability in adults (1), encompassing both cerebral hemorrhage and cerebral infarction. In the case of cerebral infarction, blood flow to a specific region of the brain is interrupted, leading to the progressive death of oxygen-deprived brain cells. Conversely, cerebral hemorrhage results from the rupture of intracranial blood vessels, allowing blood to infiltrate or accumulate within the brain tissue. Upon hemorrhage, the accumulated blood forms a hematoma that exerts pressure on the surrounding neural tissue. Simultaneously, the disruption of blood supply prevents adequate perfusion to the affected area, resulting in rapid neuronal death due to hypoxia and exposure to toxic substances, such as byproducts of hemoglobin degradation (2). The death of these cells can result in partial or complete loss of functions associated with that region, such as memory and muscle control. The impact of a stroke on an individual depends on where in the brain the stroke occurs and the extent of brain damage. When the damage occurs in a motor area of the brain, it can lead to severe motor deficits, especially in the upper extremities. Upper limb weakness and functional impairment are major challenges for survivors, and approximately 80% of stroke patients experience persistent upper limb movement disorders that require therapeutic intervention (3). Although various rehabilitation techniques are available to restore motor function, their effectiveness varies significantly among patients. Therefore, it is crucial to develop the individualized rehabilitation plan for each patient and accurately predict their potential motor recovery. A biomarker can be defined as “an indicator of disease state that can be used to measure potential molecules or cells that may be difficult to measure directly in humans” (3, 4). Biomarkers provide clinicians with the means to identify recovering patients with recovery potential, design personalized rehabilitation plans, and set realistic goals and expectations for patients and their families (5).

Recently, there has been increasing evidence supporting the use of biomarkers to predict motor recovery after stroke, including applied tools for neurophysiological and neuroimaging assessments. In the initial months following a stroke, functional recovery is primarily driven by the reorganization of brain circuits and neuroplasticity [the morphological and functional adaptation of the nervous system (6)]. Consequently, brain dynamics indicators associated with motor outcomes are highly relevant for characterizing patient profiles, serving not only as potential biomarkers but also as components of multimodal assessments that offer insights into post-stroke recovery mechanisms. Indeed, electroencephalogram (EEG) measurements performed after stroke onset can capture the neural reorganization underlying clinical recovery by detecting alterations in interhemispheric balance, activity changes within affected brain regions, and modifications in somatosensory representation maps (6, 7). Specifically, EEG can be integrated into assessment protocols that correlate cortical electrical activity changes with clinically rated neurological impairments, highlighting its promising utility in rehabilitation research.

Measuring the relationship between the severity of upper limb motor injury and cortical activity by EEG is of great value for gaining insight into cortical recombination during stroke recovery (8). Resting-state EEG is widely used for functional assessment after stroke, and the intensity of spontaneous neuronal oscillations in different frequency bands has emerged as a potential neurobiomarker associated with stroke injury and recovery. Several frequency bands commonly used include *δ*, *θ*, *α*, *β*, and *γ*. Ramanathan et al. (9) recently found that task-related low-frequency activity in the motor cortex is a marker of motor control recovery in both rodents and humans, and attenuates early after stroke and is later associated with motor function recovery. Improved brain function is associated with improved clinical symptoms (10). However, there is limited research on the longitudinal association between clinical improvement and changes in cortical activation (4, 11). It is important to note that motor recovery is a complex process that is influenced not only by spontaneous recovery, but also by intervention-induced neuroplasticity. The extent of cortical deficits after stroke can be quantified by resting state electroencephalography, as changes in resting state cortical activity are associated with motor dysfunction (12). It has been found that stroke is associated with increased low-frequency brain oscillations in *δ* (0.5–4 Hz) and *θ* (4–8 Hz) bands (13, 14), and decreased *α* (8–12 Hz) activity (15). Unilateral stroke may also affect the activity of cortical areas by altering the spectral power distribution in the hemisphere. The Brain Symmetry Index (BSI) compares the power spectra between the two hemispheres of the brain, providing a measure of their asymmetrical amplitude. It is one of the more widely used electroencephalographic-derived parameters in the field of stroke prediction. Initially developed for early detection of cerebral ischemia during carotid surgery (16), it has now been inferred to assess ischemic changes following a stroke. This observed asymmetry can be quantified and appears to correlate with the severity of stroke (17). This observed asymmetry can be quantified (18), and appears to correlate with the severity of stroke (14, 19). Recent analyses have shown that BSI calculated in the *δ* band is longitudinally correlated with FM-UL, while BSI, δ band (BSIδ), and *θ* band (BSIθ) is longitudinally correlated with NIHSS. However, the potential of EEG parameters as additional prognostic biomarkers when combined with clinical scores of upper limb motor recovery after stroke, remains unknown.

In contrast to biomarkers that predict the natural course of stroke recovery, intervention specific biomarkers have the potential to predict a patient’s response to a specific treatment. This approach holds great promise for identifying the most appropriate patients for recovery in a clinical setting. Vatinno et al. (20) investigated sensorimotor connectivity to predict upper limb motor recovery after stroke task training. Zhou et al. (21) used EEG to measure parietal lobe (PF) network connectivity to predict motor gain in visuospatial training after stroke. Previous studies have demonstrated the utility of EEG as a predictor of intervention-related motor gain at different time points before and after the intervention. However, it is important to note that EEG signals are non-stationary, complex, and heavily influenced by the stage and timing of stroke recovery.

Therefore, we propose integrating routine rehabilitation interventions with EEG signal acquisition to analyze changes in EEG signals and brain networks during the rehabilitation process. This involves regularly assessing the brain’s response to rehabilitation interventions throughout the treatment course. At the same time, correlation analysis will be conducted with clinically relevant indicators to find out specific biomarkers, enabling the prediction of prognosis for individual patients and facilitating early intervention. Patients with poor recovery may benefit from motor relearning combined with neuropharmacological intervention to promote nerve repair. For those with moderate recovery, intensive therapeutic interventions targeting behavioral recovery can begin early. For patients demonstrating good recovery, advanced skills training (such as writing) can be intensified to further enhance functional outcomes.

After reviewing previous studies, it was found that studies on the longitudinal collection of EEG data were relatively rare. Therefore, the purpose of this study was to collect resting-state EEG longitudinally, analyze the correlation between the spectral characteristics of resting-state EEG and clinical behavioral indicators, and identify the resting-state EEG parameters related to stroke recovery; on this basis, it was also explored whether resting-state EEG has predictive value for upper limb motor impairment in patients with subacute stroke.

## 2 Method

### 2.1 Participants

**Figure 1:**
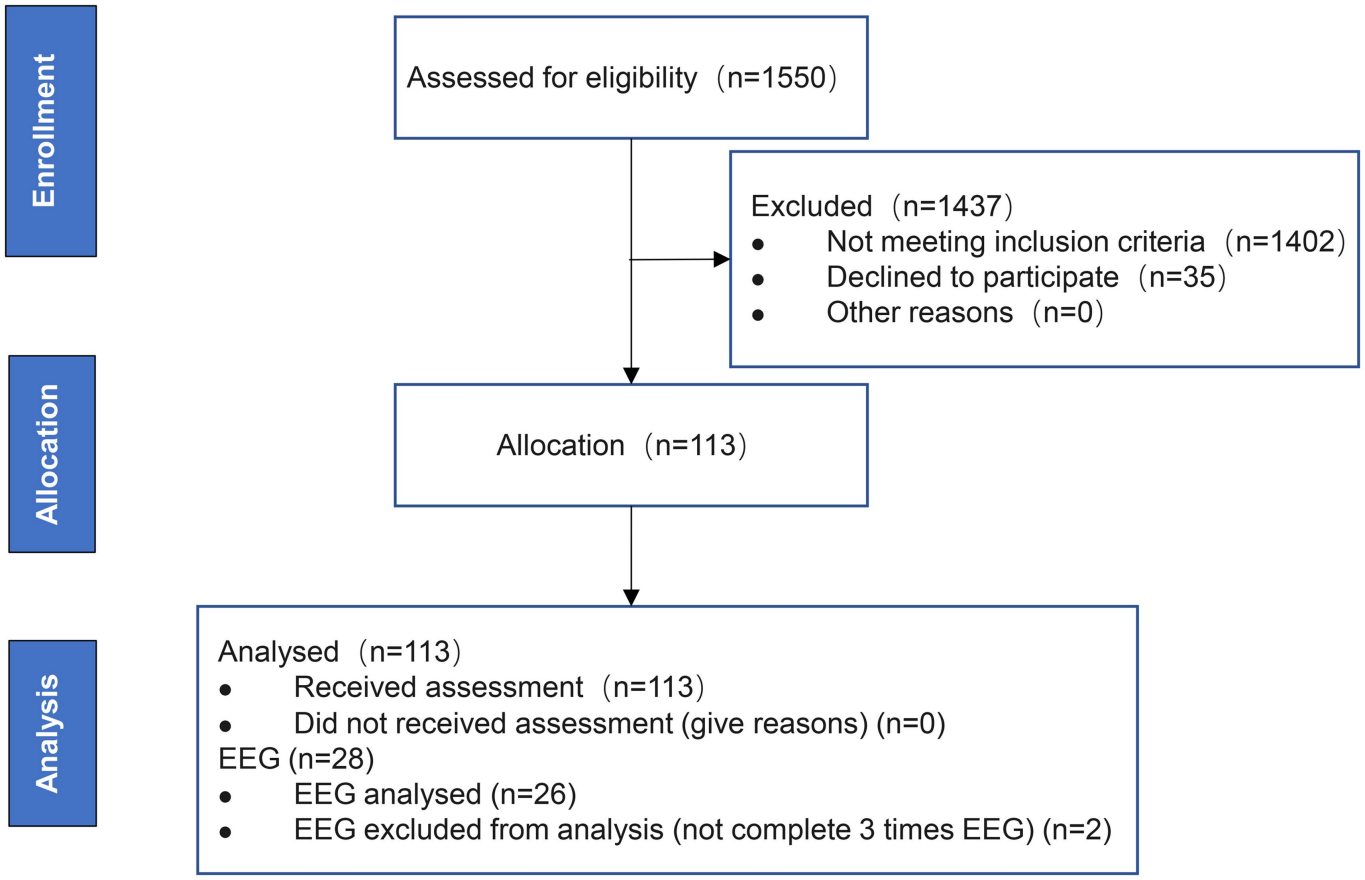
Flowchart of screening, inclusion and drop-outs.

In our multicenter longitudinal cohort study, patients admitted to the stroke units of 3 participating hospitals from June 2023 to June 2024 were eligible for participation. Among the 1,550 patients, 113 suitable patients were selected, all of whom completed the clinical baseline assessment and the assessment after 2 weeks. Among them, 26 patients completed the EEG recording at baseline and completed the EEG recording at 1 and 2 weeks of recovery respectively, and were included in the analysis. A flowchart of screening, inclusion and drop-outs is depicted in Figure 1.

The inclusion criteria were: (1) first-ever stroke according to computed tomography or magnetic resonance imaging (MRI) scan; (2) age from 18 to 80 years; (3) at least 2 weeks since stroke onset and less than 6 month; (4) able to sit on a chair independently for at least 1 h; (5) stable vital signs, able to complete routine post-stroke rehabilitation assessment (6) signed informed consent and volunteered to participate in this study. Exclusion criteria: (1) the presence of any disease or symptom (secondary stroke, fall, fracture) that may aggravate or cause adverse effects of exercise; (2) patients with cognitive impairment (Mini-Mental State Examination, MMSE < 10) or mental illness that makes them ineligible for evaluation; (3) allergic to electrode gel; (4) Patients who are unable to sign informed consent forms; (5) Patients with neurodegenerative diseases. The baseline information of 113 patients whose EEG was not collected is shown in Table 1. This study was approved by the ethical committee of Huashan Hospital [(2023) Provisional Examination No. (013)] and was performed according to the Declaration of Helsinki, and the clinical registration was conducted (ChiCTR2300068400).

**Table 1:** Baseline demographics of 113 patients.

Demographics and clinical scores	Mean (SD)
Time post stroke (days),clinical assessment	40.89 (40.74)
Age(years)	63.23 (12)
Gender(male/female)	85/28
Affected hemisphere (left/right)	43/70
Hand dominance (left/right)	1/112
Stroke type (CI/CH)	85/28

### 2.2 Behavioral indicators

Functional outcomes after stroke can be evaluated by a variety of clinical measures. According to the International Classification of Function, Disability and Health, outcome measures can be categorized into three domains: physical function and structure, activity and participation (World Health Organization, 2001). An experienced therapist performed all clinical measures. According to the changes in muscle tone and motor function, Brunnstrom divided the central nervous system injury after treatment into six stages to evaluate the recovery of motor function after nervous system injury. The Fugl-Meyer Motor Assessment of the Upper Limb (FMA-UL) is a sensitive, valid, and reliable clinical test for measuring motor function (22) of the upper limb at the injury level. The FMA-UL, an injury scale designed for stroke survivors, determines a patient’s ability to separate movement from the upper limb, is a valid predictor of upper limb movement recovery, and is recommended to most appropriately reflect “true” neuromotor recovery (23), out of a score of 66. ARAT (24) is a standardized scale designed to evaluate upper limb motor dysfunction after stroke. This scale mainly assesses the patient’s ability to operate objects of different sizes, weights and shapes with high reliability and effectiveness. There are 19 items in total, which are divided into four groups of new sub-scale items. Through 4 basic movements: grasping, gripping, pinching and gross movement, the completion quality of each task adopts a 4-level method (0 ~ 3 points), 0 indicates that the action cannot be completed, 3 indicates that the action can be completed normally; One side of the upper limb was rated on a scale of 0–57, with higher scores indicating better function. The Modified Barthel Index is used to measure activities of daily living. It is made up of 10 items related to different activities on a scale of 100, including (1) personal hygiene, (2) self-bathing, (3) eating, (4) using the toilet, (5) climbing stairs, (6) dressing, (7) defecating, (8) bladder control, (9) walking, (10) transfer. The higher the score, the better (25) the ability to perform activities of daily living. The NIHSS (26), measured hours to days after stroke onset, is an attractive outcome measure for stroke research, with NIHSS at visit (baseline NIHSS) strongly predicting subsequent NIHSS on a scale of 0–42, with higher scores indicating more severe nerve damage.

### 2.3 Routine assessment methods

**Figure 2:**
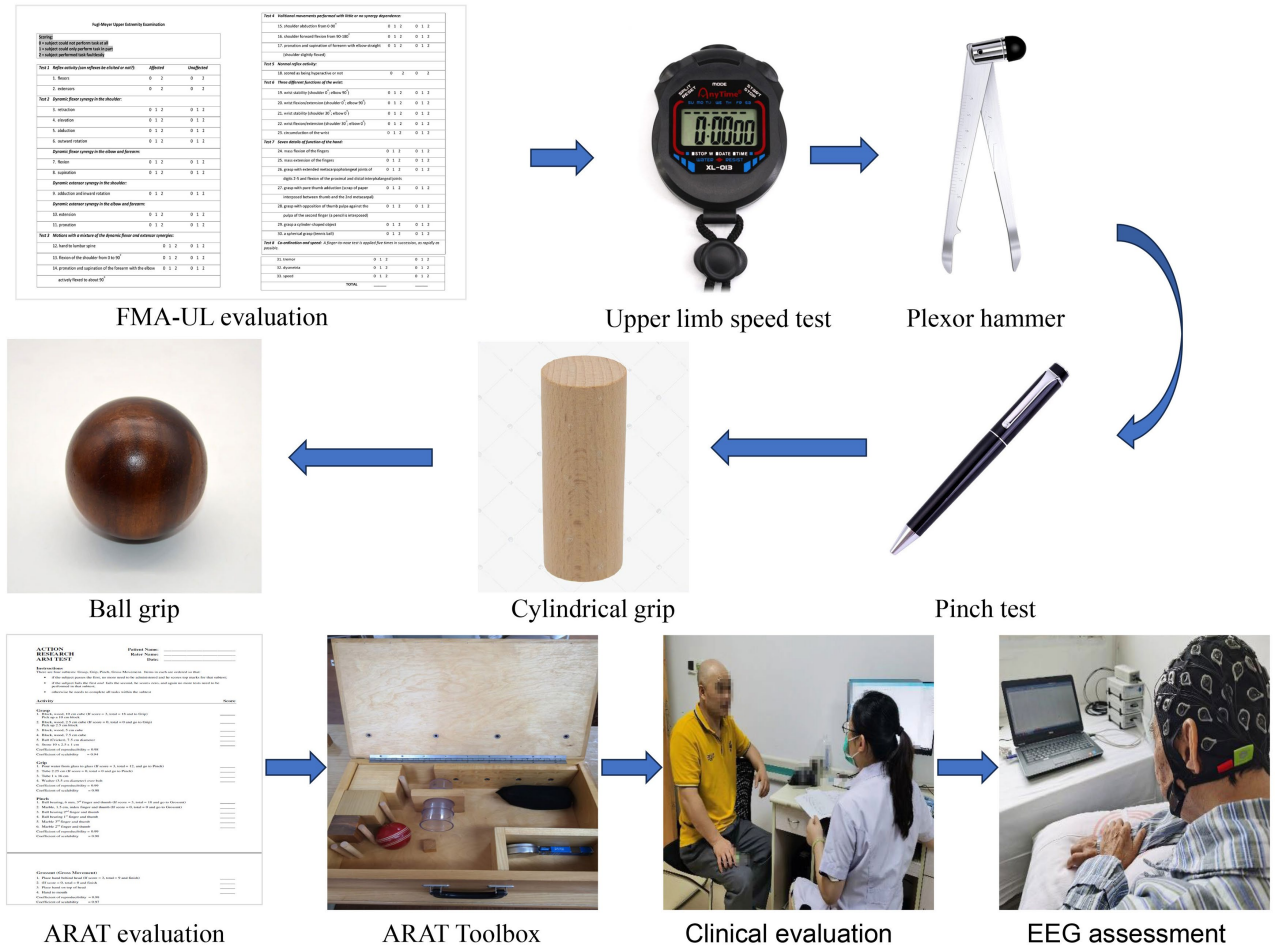
Clinical assessment and EEG assessment.

The patients were routinely treated with basic rehabilitation therapy, including exercise therapy and occupational therapy for 1 h/time, 5 times a week, for a total of 2 weeks. Clinical behavioral scales were assessed for 113 patients on day 1 (T0) and day 14 (T2) after admission. Among the 26 patients, EEG was collected using 64 channels consisting of Ag/AgCl electrode of EEG cap (actiCAP; Brain Products, Gilching, Germany) according to the configuration of 10–20 International System on the 1st day (T0), 7th day (T1), and 14th day (T2). The signal is amplified by the amplifier (Brain Products). The reference electrode was located in the right mastoid process, and the ground electrode was located in the forehead. The electrode impedance was kept below 20 kΩ. The original EEG signals was recorded at a sampling rate of 500 Hz and filtered by a bandpass filter between 0.1 and 60 Hz. Subjects kept relaxed and motionless during the experiment. The experimental environment was kept in weak light, quiet, and with no electromagnetic interference. Clinical assessments are conducted on the 1st and 14th days simultaneously. The patients were asked to remain quiet while their EEG was collected for 10 min. The upper limb of Fugl-Meyer assessment, ARAT, NIHSS and MBI were collected before (T0) and after the 2-weeks intervention (T2). EEG assessment and clinical evaluations were performed in a quiet room for all patients (Figure 2).

### 2.4 The analysis process of resting EEG

**Figure 3:**
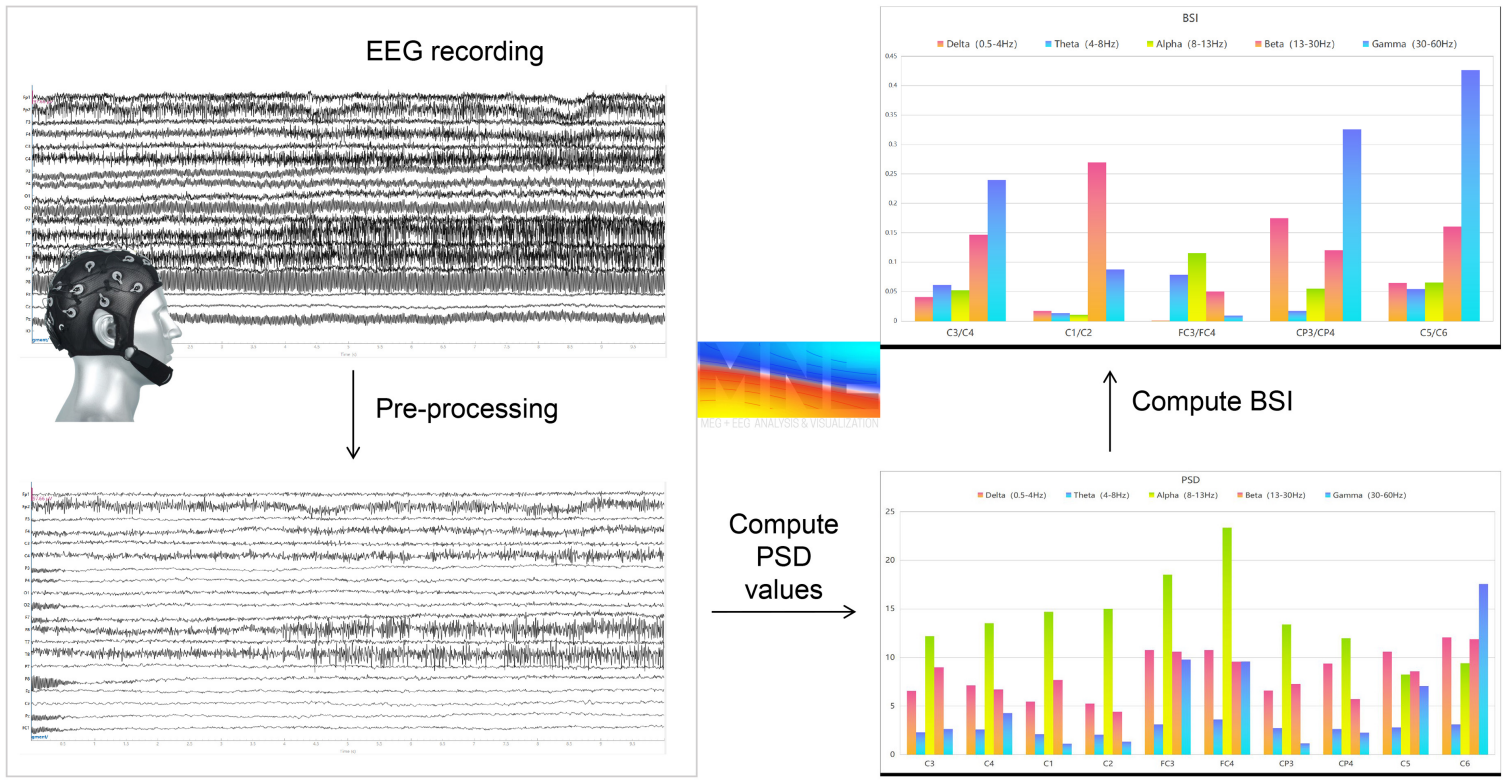
Schematic diagram of computing several brain indexes based on raw EEG data using MNE-Python.

Offline analysis was conducted using MNE-Python software (27, 28). Refer to the previous research of the research group, the left hemisphere was covered with FC3, CP3, C1, C3, and C5 (five channels) while the right with FC4, CP4, C2, C4, and C6 (five channels) (29). Firstly, load the raw data into MNE-Python. Then, load the electrode channel positions of the EEG data and delete the IO channel. Then use a notch filter to remove the 50 Hz power frequency and perform a 0.1 Hz- 60 Hz bandpass filter. Then, run an independent component analysis and draw the ICA component topography. Remove the electrooculogram component according to the ICA component topography, select the number of the component to be deleted, delete the component and apply it to the EEG data. Then perform the re-reference: apply the whole brain average reference. Finally, perform frequency band division (delta: 0.5-4 Hz, theta: 4-8 Hz, alpha: 8-13 Hz, beta: 13-30 Hz, gamma: 30-60 Hz) and compute Power spectrum (PSD) of each band. The brain symmetry index (BSI) was calculated and exported. Figure 3 shows the schematic diagram of computing several brain indexes based on raw EEG data using MNE-Python.

#### 2.4.1 Electrode channels

High-density 64-channel EEG recording using active shielding EEG cap, electrode placement in accordance with international standard 10–20 system, the sampling rate of 2048KZ (Ag/AgCl electrodes and REFA multichannel amplifier, Netherlands, with ASA acquisition software, ANT Software BV, The Netherlands), the electrodes located in the mastoid process (m1, m2) were not used, resulting in 62 channel recording. The ground electrode was placed on the mastoid. Record the signal as an average reference. Resting-state EEG with eyes open was acquired while subjects were seated and focused their eyes on a computer screen for 10 min. Keep the electrode impedance below 20 kΩ. The EEG signal is referred to the average online.

#### 2.4.2 Division of frequency bands

To facilitate analysis from clinical and cognitive scientific perspectives, EEG signals are divided into several frequency ranges. They are *δ* (0.5–4 Hz), *θ* (4–8 Hz), *α* (8–13 Hz), *β* (13–30 Hz), and *γ* (30–60 Hz). Delta band EEG is a low-frequency, high-amplitude wave associated with slow-wave sleep (SWS). SWS, in turn, is associated with long-term memory (30). In the adult brain, delta activity originates in the frontal cortex, hippocampus and thalamus. Human *θ*-band EEGs may come from either the cortex or the hippocampus, and the two forms may be independent of each other. In addition, θ waves are associated with memory processes in our brains. Interneurons in the hippocampus and prefrontal cortex essentially oscillate (31) at θ band frequencies.

Alpha waves originate in the posterior part, with the largest portion in the anterior part, especially in the occipital lobe, and may be more widely distributed. The power of the alpha frequency increases from early childhood to adulthood, but decreases with age or age-related neurological disorders. In people with dementia, as well as many other types of neurological disorders, the amplitude of the alpha frequency decreases, and it is significant in subjects with good memory.

In general, beta EEG is associated with our waking state, and specifically with cognitive and emotional processes. Between the two brain hemispheres, beta wave amplitudes in the brain hemispheres can vary by up to 35% (19).

Gamma rhythms occur after sensory stimulation in humans and other mammals, and are usually short-lived. Gamma rhythms have been identified in the sensory cortex as well as in the hippocampus. In addition to sensory information processing, perceptual gamma electroencephalography is involved in memory formation, language processing, internal thoughts (without any external stimuli), and behavior, especially motor movements and action plans.

#### 2.4.3 BSI–brain symmetry index

BSI is defined as the absolute pairwise normalized spectral power difference between the left and right homologous channels cL and cR. In the frequency range of 0.5 to 60 Hz, we considered the absolute value of the relative difference in the average spectral density between the left and right hemispheres as a measure of symmetry. Since the spectral density is estimated via the Fast Fourier Transform, we now define the Brain Symmetry Index as follows (32):


$${\mathrm{BSI}}_{C}={\langle |\frac{P_{C_{R}}(f)-P_{C_{R}}(f)}{P_{C_{R}}(f)+P_{C_{R}}(f)}|\rangle }_{f=1,\ldots ,25\mathrm{Hz}}$$

These values were averaged over all channel pairs *cp*:


$$\mathrm{BSI}=\frac{2}{N}\sum_{\mathrm{cp}=1}^{N/2}{\mathrm{BSI}}_{\mathrm{cp}}$$

BSI has an upper bound of 1, reflecting the maximum asymmetry of all paired channels; The lower bound is zero, indicating perfect symmetry. The electrodes on the middle line are excluded because they do not form a symmetrical channel. When one electrode with a symmetrical channel is considered a bad channel, the corresponding paired channel is excluded.

## 3 Statistical methods

According to FMA-UL, ARAT, MBI, NIHSS, as well as BSI and PSD EEG indicators, normality and homogeneity of variance tests were performed, ultimately leading to the selection of non-parametric statistical methods. Descriptive statistical results were expressed by median ± interquartile range. Spearman correlation analysis was used in the correlation analysis of statistical inference results. Regression analysis was performed between different times of clinical scales, between PSD as well as BSI and clinical scales. Repeated measures analysis of variance (RM-ANOVA) was performed between PSD and BSI in T0, T1 and T2. *p* < 0.05 was considered statistically significant.

## 4 Results

### 4.1 Behavioral scale results

Table 2 showed that the two behavioral scale evaluations on Day 1 (T0) and Day 14 (T2) of the 113 patients were positively correlated, suggesting that the better the baseline motor function of patients, the better the later recovery. The score of the scale has a high predictive effect on the functional prognosis of patients in the later period.

**Table 2:** Changes, correlations and regression analysis of functional scores before and after treatment (*n* = 113).

Project	Pre-treatment	Post-treatment	Pre-post change		Correlation		Regression		
			Z	*p*	ρ	*p*	B(SE)	*R*^2^	*p*
FMA-UL	31.2 ± 20.7	39.3 ± 21.4	−8.475	<0.001	0.618	<0.001	0.929 (0.043)	0.811	<0.001
ARAT	19.1 ± 23.3	27 ± 25.2	−6.112	<0.001	0.924	<0.001	0.935 (0.052)	0.744	<0.001
MBI	52.8 ± 24.7	66.4 ± 22.9	−8.371	<0.001	0.865	<0.001	0.792 (0.046)	0.730	<0.001
NIHSS	4.2 ± 3.4	3.1 ± 3.1	−5.970	<0.001	0.807	<0.001	0.781 (0.043)	0.750	<0.001

FMA-UL, upper limb part of Fugl-Meyer scale.

In order to further verify which EEG spectrum was associated with the improvement of motor function under conventional intervention, we further collected EEG for 26 patients three times on Day 1 (T0), Day 7 (T1) and Day 14 (T2) of admission. At the same time, the behavioral scale was assessed on Day 1 (T0) and Day 14 (T2), respectively, as in the previous 113 patients. Similarly, the 26 patients were positively correlated with the two behavioral scales on Day 1 and Day 14, as shown in Table 3.

**Table 3:** Changes, correlations and regression analysis of functional scores before and after treatment (*n* = 26).

Project	Pre-treatment	Post-treatment	Pre-post change		Correlation		Regression		
			Z	*p*	ρ	*p*	B(SE)	R^2^	*p*
FMA-UL	32.8 ± 17.2	40.2 ± 20.1	−4.290	<0.001	0.929	<0.001	1.106 (0.075)	0.901	<0.001
ARAT	19.7 ± 23.6	28.2 ± 25.5	−3.182	<0.001	0.923	<0.001	0.930 (0.111)	0.744	<0.001
MBI	68.3 ± 21.1	79 ± 17.6	−4.380	<0.001	0.912	<0.001	0.742 (0.077)	0.796	<0.001
NIHSS	2.7 ± 2.1	2.1 ± 2.2	−2.911	0.004	0.825	<0.001	0.918 (0.097)	0.790	<0.001

FMA-UL, upper limb part of Fugl-Meyer scale.

**Figure 4:**
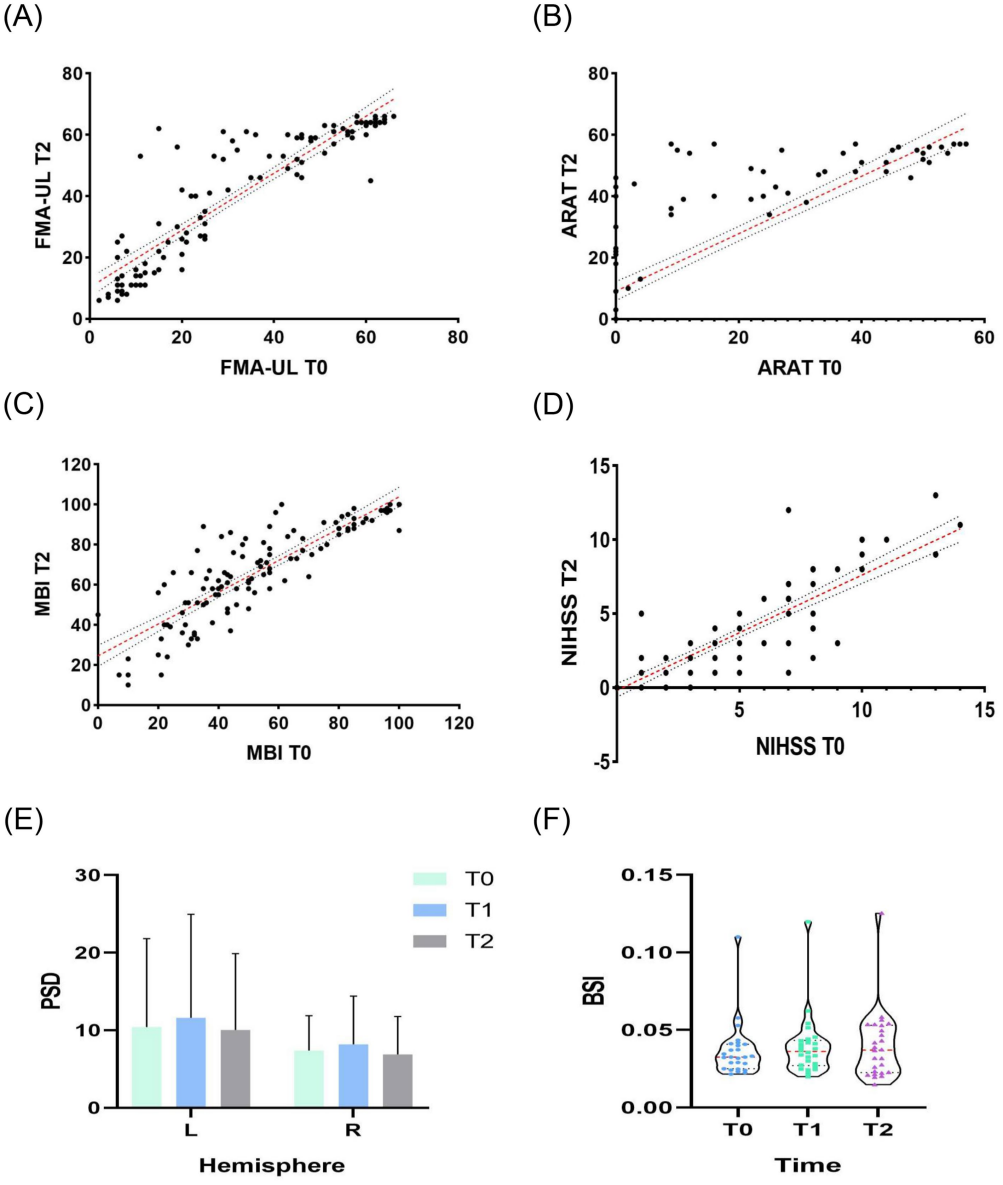
Regression analysis of 113 patients in T0 and T2, beta PSD of left and right hemispheres in T0, T1 and T2, and violin plots of BSI in T0, T1 and T2. L, left; R, right; T0, week 0; T1, week 1; T2, week 2.

Figure 4 shows the regression results of 113 patients in T0 and T2 (Figures 4A–D, details see Table 2), beta PSD of left and right hemispheres in T0, T1 and T2 (Figure 4E), and violin plots of beta BSI in T0, T1 and T2 (Figure 4F). For beta BSI, one-way ANOVA showed no significant differences among groups [*F*(2, 75) = 0.18, *p* = 0.832, η^2^ = 0.005]. One-way ANOVA revealed no significant group differences in left-hemisphere PSD [*F*(2, 75) = 0.13, *p* = 0.879, η^2^ = 0.003], with homogeneity of variance confirmed by Brown-Forsythe (*p* = 0.792) and Bartlett’s tests (*p* = 0.322), indicating stable measures across conditions (*N* = 78). Similarly, right-hemisphere PSD showed no significant between-group differences [*F*(2, 75) = 0.40, *p* = 0.669, η^2^ = 0.011], supported by homogeneous variance (Brown-Forsythe: *p* = 0.571; Bartlett’s: *p* = 0.237), demonstrating consistent measures (*N* = 78). The negligible to small effect sizes (left: *R*^2^ = 0.003; right: *R*^2^ = 0.011) further confirmed minimal between-group variability in both hemispheres. Variance homogeneity was confirmed (Brown-Forsythe: *p* = 0.906; Bartlett’s: *p* = 0.868). The effect size was negligible (*R*^2^ = 0.009), indicating minimal between-group variability (total *n* = 78). BSI measures were consistent across conditions.

### 4.2 Correlation analysis results

#### 4.2.1 PSD

We also test whether the relative powers in each frequency band were correlated with the Brunnstrom, FMA-UL, ARAT, MBI and NIHSS scales. We only found a correlation in the *β* band. There is a positive relationship between PSD T1 and FMA-UL T2 (*r* = 0.469, *p* = 0.016), PSD T2 and FMA-UL T2 (*r* = 0.391, *p* = 0.048), while no relationship between PSD T0 and FMA-UL T0/T2. There was still a positive correlation between PSD T0/T1/T2 and FMA-HAND T0/T2. ARAT T0/T2 was also positively correlated with PSDT0/T1/T2 (see Table 4).

**Table 4:** Correlation analysis between scales at week 0 and week 2, and EEG PSD in beta band (*n* = 26).

Subgroup	T0 scales with first PSD		T2 scales with first PSD		T2 scales with second PSD		T2 scales with third PSD	
	*r*	*p*	r	*p*	r	*p*	r	*p*
Brunnstrom-UL	0.352	0.077	0.254	0.210*	0.425	0.03*	0.319	0.112
Brunnstrom-H	0.438	0.025*	0.367	0.066	0.421	0.032*	0.452	0.02*
FMA-UL	0.358	0.073	0.342	0.087	0.469	0.016*	0.391	0.048*
FMA-HAND	0.44	0.024*	0.455	0.019*	0.518	0.007*	0.455	0.019*
ARAT	0.435	0.026*	0.452	0.02*	0.428	0.029*	0.409	0.038*
MBI	0.199	0.329	0.289	0.153	0.399	0.043*	0.37	0.063
NIHSS	−0.164	0.425	−0.198	0.333	−0.396	0.045*	−0.171	0.403

* *p* < 0.05. Brunnstrom-UL, upper limb part of Brunnstrom scale; Brunnstrom-H, hand part of Brunnstrom scale; FMA-UL, upper limb part of Fugl-Meyer scale; FMA-HAND, hand part of Fugl-Meyer scale.

#### 4.2.2 BSI index

We test whether the relative powers in BSI were correlated with the Brunnstrom, FMA-UL, ARAT, MBI and NIHSS scales. We found a negative relationship between BSI T2 and Brunnstrom UL T2 (*r* = −0.546, *p* = 0.004), while no relationship between BSI T0/T1 and Brunnstrom UL T0/T2. There is a negative relationship between BSI T1 and FMA-UL T2 (*r* = −0.443, *p* = 0.023), BSI T2 and FMA-UL T2 (*r* = −0.619, *p* = 0.001), while no relationship between BSI T1 and FMA-UL T2. There is a negative relationship between BSI T1 and FMA-HAND T2 (*r* = −0.46, *p* = 0.018), BSI T2 and FMA-HAND T2 (*r* = −0.608, *p* = 0.001), while no relationship between BSI T0/T1and FMA-HAND T0/T2. There is a negative relationship between BSI T1 and ARAT T2 (*r* = −0.504, *p* = 0.009), BSI T2 and ARAT T2 (*r* = −0.554, *p* = 0.003), while no relationship between BSI T0 and ARAT T0/T2. There is a negative relationship between BSI T2 and MBI T2 (*r* = −0.568, *p* = 0.002), while no relationship between BSI T0/T1 and MBI T0/T2. There is a positive relationship between BSI T2 and NIHSS T2 (*r* = −0.558, *p* = 0.003), while no relationship between BSI T0/T1 and NIHSS T0/T2 (see Table 5).

**Table 5:** Correlations between BSI in beta band and Brunnstrom, FMA-UL, ARAT, MBI and NIHSS scales (*n* = 26).

Subgroup	T0 scales with first BSI		T2 scales with first BSI		T2 scales with second BSI		T2 scales with third BSI	
	*r*	*p*	*r*	*p*	*r*	*p*	*r*	*p*
Brunnstrom-UL	−0.361	0.07	−0.233	0.251	−0.292	0.147	−0.546	0.004*
Brunnstrom-H	−0.173	0.399	−0.182	0.373	−0.489	0.066	−0.477	0.014*
FMA-UL	−0.405	0.04*	−0.282	0.163	−0.443	0.023*	−0.619	0.001*
FMA-HAND	−0.355	0.075	−0.25	0.217	−0.46	0.018*	−0.608	0.001*
ARAT	−0.352	0.078	−0.343	0.087	−0.504	0.009*	−0.554	0.003*
MBI	−0.356	0.074	−0.358	0.072	−0.299	0.138	−0.568	0.002*
NIHSS	0.456	0.019*	0.367	0.065	0.369	0.063	0.558	0.003*

*p < 0.05. Brunnstrom-UL, upper limb part of Brunnstrom scale; Brunnstrom-H, hand part of Brunnstrom scale; FMA-UL, upper limb part of Fugl-Meyer scale; FMA-HAND, hand part of Fugl-Meyer scale.

### 4.3 Regression analysis results

For PSD, our regression analysis indicated that baseline PSD-T0 significantly predicted only FMA-Hand scores at follow-up (*β* = 0.997, 95% CI [0.22–1.79], *p* = 0.014), accounting for 22.8% of the variance in hand motor function. With respect to broader motor outcomes, PSD-T0 showed consistent positive trends: higher baseline power spectral density predicted better Brunnstrom-Hand (*β* = 0.163, *p* = 0.041), FMA-UL (*β* = 1.787, *p* = 0.043), and ARAT (*β* = 2.416, *p* = 0.030) performance, although the association with Brunnstrom-UL did not reach statistical significance (*β* = 0.080, *p* = 0.134). These findings suggest that elevated left-hemisphere PSD at baseline may herald more favorable functional recovery. Moreover, regression analyses revealed that follow-up PSD-T1 exhibited stronger and more widespread predictive relationships than baseline measurements. PSD-T1 demonstrated significant associations with Brunnstrom-UL (*β* = 0.088, 95% CI [0.02–0.16], *p* = 0.017), Brunnstrom-Hand (*β* = 0.145, 95% CI [0.04–0.25], *p* = 0.010), FMA-UL (*β* = 1.624, 95% CI [0.45–2.80], *p* = 0.009), FMA-Hand (*β* = 0.788, 95% CI [0.25–1.33], *p* = 0.006), and ARAT (*β* = 1.945, 95% CI [0.43–3.46], *p* = 0.014), collectively explaining 21.4–27.3% of outcome variance. While MBI and NIHSS displayed consistent directional trends, these relationships were not statistically significant (MBI: *β* = 0.907, *p* = 0.109; NIHSS: *β* = −0.131, *p* = 0.062). Collectively, the emergence of significant predictions across multiple motor domains at follow-up underscores that PSD measurements acquired during active rehabilitation possess enhanced prognostic utility relative to baseline assessments (see Table 6).

**Table 6:** Regression analysis between PSD measures in beta band and clinical outcomes (*n* = 26).

Predictor	PSD-T0 (Baseline)				PSD-T1 (Follow-up)			
T2 scales	*β* (SE)	95% CI	*R*^2^	*p*	*β* (SE)	95% CI	*R*^2^	*p*
NIHSS	−0.127 (0.096)	−0.33, 0.07	0.068	0.2	−0.131 (0.067)	−0.27, 0.01	0.138	0.062
Brunnstrom-UL	0.080 (0.051)	−0.03, 0.19	0.091	0.134	0.088 (0.034)*	0.02, 0.16*	0.214	0.017*
Brunnstrom-H	0.163 (0.075)*	0.01, 0.32*	0.163	0.041*	0.145 (0.051)**	0.04, 0.25**	0.25	0.010**
FMA-UL	1.787 (0.836)*	0.06, 3.51*	0.16	0.043*	1.624 (0.570)**	0.45, 2.80**	0.253	0.009**
FMA-HAND	0.997 (0.374)**	0.22, 1.79**	0.228	0.014*	0.788 (0.262)**	0.25, 1.33**	0.273	0.006**
ARAT	2.416 (1.047)*	0.26, 4.58*	0.182	0.030*	1.945 (0.736)*	0.43, 3.46*	0.226	0.014*
MBI	0.625 (0.788)	−1.00, 2.25	0.026	0.436	0.907 (0.546)	−0.22, 2.03	0.103	0.109

*p < 0.05, **p < 0.01. Brunnstrom-UL, upper limb part of Brunnstrom scale; Brunnstrom-H, hand part of Brunnstrom scale; FMA-UL, upper limb part of Fugl-Meyer scale; FMA-HAND, hand part of Fugl-Meyer scale.

For BSI, our regression analysis demonstrated that baseline BSI (BSIT0) significantly predicted NIHSS scores at follow-up (*β* = 48.63, 95%CI [0.07–97.19], *p* = 0.0497*), explaining 15.1% of the variance in neurological impairment. For motor outcomes, BSIT0 showed consistent predictive trends: higher baseline asymmetry predicted poorer Brunnstrom-UL (*β* = −32.26, *p* = 0.1194) and FMA-UL (*β* = −174.8, *p* = 0.1027) performance, though these associations did not reach statistical significance. This pattern suggests that greater interhemispheric imbalance at baseline may forecast less favorable functional recovery. Besides, regression analysis revealed that follow-up BSI (BSIT1) showed stronger predictive relationships than baseline measurements, demonstrating significant associations with Brunnstrom-Hand (*β* = −401.7, 95%CI[−802.8 to −0.6], *p* = 0.0497*) and FMA-UL (*β* = −194.4, 95%CI[−387.5 to −1.3], *p* = 0.0413*), collectively explaining 15.1–18.2% of outcome variance. While other motor scales showed consistent directional trends (ARAT: *β* = −214.6, *p* = 0.243; MBI: *β* = −16.96, *p* = 0.169), these relationships were not statistically significant. Notably, the emergence of significant predictions for hand motor function at follow-up suggests that BSI measurements during rehabilitation may have enhanced clinical prognostic value compared to baseline assessments (see Table 7).

**Table 7:** Regression analysis between BSI measures in beta band and clinical outcomes (*n* = 26).

Predictor	BSIT0 (Baseline)				BSIT1 (Follow-up)			
T2 scales	*β* (SE)	95% CI	*R*^2^	*p*	*β* (SE)	95% CI	*R*^2^	*p*
NIHSS	48.63 (23.53)	0.07, 97.19	0.151	0.0497*	36.48 (21.84)	−7.56, 80.52	0.104	0.1079
Brunnstrom-UL	−32.26 (19.96)	−73.49, 8.67	0.096	0.1194	−39.73 (17.19)	−75.20, −4.26	0.182	0.0297*
Brunnstrom-H	−395.5 (219.0)	−847.5, 86.53	0.117	0.0835	−401.7 (194.3)	−802.8, −0.6	0.151	0.0497*
FMA-UL	−174.8 (103.0)	−387.5, 37.84	0.107	0.1027	−194.4 (90.18)	−387.5, −1.3	0.162	0.0413*
FMA-HAND	−523.8 (276.2)	−1,004, 46.34	0.13	0.07	−464.1 (250.3)	−980.7, 52.5	0.125	0.0761
ARAT	−306.9 (194.4)	−706.1, 64.29	0.094	0.1275	−214.6 (179.3)	−584.2, 155.0	0.056	0.243
MBI	−19.71 (13.18)	−46.90, 7.47	0.085	0.1178	−16.96 (11.96)	−41.63, 7.71	0.077	0.169

*p < 0.05. Brunnstrom-UL, upper limb part of Brunnstrom scale; Brunnstrom-H, hand part of Brunnstrom scale; FMA-UL, upper limb part of Fugl-Meyer scale; FMA-HAND, hand part of Fugl-Meyer scale.

## 5 Discussion

After a stroke, molecules that promote the remodeling of dendritic, axonal, and synaptic structures increase, while those that inhibit axonal plasticity decrease. Surviving neurons are capable of growing new axons and synapses; following selection and pruning processes, some neural connections in the penumbra and other denervated brain regions may be partially restored (33, 34). Rehabilitation training not only improves blood flow around the infarcted area and promotes angiogenesis but also protects the blood–brain barrier, thereby preserving neuronal function in the vicinity of the infarct (35, 36). Additionally, rehabilitation training can alter neuronal structure by promoting neurogenesis after brain injury as well as neuron differentiation and survival while inhibiting apoptosis. It also involves pruning dendrites around the infarcted area and regulating synaptic plasticity (36, 37), providing a morphological basis for sustained functional recovery. Exercise training enhances cortical plasticity on the affected side by increasing dendritic pruning and synaptic remodeling around the ischemic region while simultaneously elevating astrocyte numbers (37). Imaging studies [such as positron emission tomography (PET), electroencephalography (EEG), and functional magnetic resonance imaging (fMRI)] have shown extensive changes in brain activation patterns when simple movements are performed with the affected hand post-stroke; these temporal changes align with gradual reorganization within sensory-motor systems. In the affected hemisphere, activation of primary motor cortex (M1) decreases; depending on both location and extent of ischemic damage, its activity may shift to more posterior (38) or anterior areas (39), particularly toward regions such as supplementary motor area (SMA) that are typically unaffected by standard middle cerebral artery occlusion, particularly toward regions such as supplementary motor area (SMA) that are typically unaffected by standard middle cerebral artery occlusion (40).

Currently, there exist two predictive models regarding interhemispheric competition: “the substitution model” and “the interhemispheric competition model.” The former is based on research utilizing transcranial magnetic stimulation to disrupt motor region functionality which suggests that activity from an unaffected hemisphere may facilitate functional recovery post-stroke; under this model residual network activity compensates for lost functions in damaged areas. This process likely occurs entirely within an intact hemisphere. Conversely, “the interhemispheric competition model” posits a reciprocal inhibitory balance between both hemispheres in a healthy brain. When one hemisphere suffers damage due to stroke, this balance is disrupted leading to diminished inhibition exerted by the affected hemisphere upon its unaffected counterpart resulting in enhanced inhibition from it instead. Consequently arises what is termed “dual impairment,” where ipsilateral damage combines with excessive contralateral suppression (41). The two proposed recombination models provide opposing predictions regarding whether the optimal neuroregulatory treatment for individual patients should primarily focus on inhibition or excitation. However, we contend that both the “substitution model” and the “interhemispheric competition model” exhibit significant shortcomings. Electroencephalography (EEG), as an effective and cost-efficient method, can be utilized to assess the state of residual brain regions and assist in selecting non-invasive brain stimulation (NIBS) protocols. Furthermore, it enables exploration of brain activity and connectivity during resting states, providing immediate information for evaluating cortical tissue functional integrity. Research has indicated that symmetrical spectral power between hemispheres is associated with mild neurological deficits (42), while increased asymmetry suggests acute deterioration and poor prognosis (13, 43). The results revealed that BSI was closely associated with functional recovery.

Studies have shown that EEG indicators provide valuable information for predicting functional recovery in patients with acute stroke (13, 44), and they help to distinguish patients with acute stroke from healthy controls (45). On the other hand, its effectiveness in the subacute phase of stroke remains poorly understood. Several studies have also investigated the predictive relationship between brain electrical frequency and behavioral data. Research has demonstrated a significant correlation between EEG activity monitored in real-time during fixed upper limb movements and various clinical measures, including the Fugl-Meyer upper limb score, movement duration, smoothness metrics, and peak velocity. Furthermore, EEG measurements indicate that patients with better clinical conditions exhibit a tendency for the healthy hemisphere to compensate for the affected hemisphere. This finding underscores the important relationship between kinematic indicators—particularly movement duration and smoothness—and EEG biomarkers in assessing recovery following a stroke (46). BSI, particularly in the theta and *δ* bands, correlated with motor recovery after stroke. A reduction in BSI-delta reflected an improvement in overall nerve damage and was also particularly associated with early upper limb motor recovery after stroke (18). However, no studies have been conducted on the distribution of the power spectrum of spontaneous EEG in the subacute phase with conventional rehabilitation interventions. In patients with poor motor function, brain activity appeared in bilateral brain regions, while in patients with good motor function, brain activity was limited to the ipsilateral region.

In addition, quantitative EEG analysis is becoming a standardized procedure, thus guaranteeing repeatability of EEG results from different centers. Furthermore, EEG measurements can capture a wealth of information on neural activity including oscillations (PSD), neural interactions (connectivity), networks and spatial distribution (BSI). Therefore, EEG is undoubtedly a general-purpose tool with which many clinicians are becoming increasingly familiar with quantitative analysis. Biomarkers hold the potential to provide valuable information about patients, enabling researchers and clinicians to identify specific biological subgroups and/or those that may benefit from a treatment approach. Given the heterogeneity of stroke, biomarkers have the potential to significantly impact the field of stroke rehabilitation. Previous neurophysiological studies have revealed that low-frequency oscillations in resting-state EEG recordings from stroke patients can reflect both the extent of neural damage and subsequent recovery processes. These oscillatory patterns may serve as potential biomarkers for evaluating post-stroke rehabilitation outcomes (47). Furthermore, cortical motor coherence (CMC) has emerged as an effective neurophysiological approach for monitoring the reorganization of neural circuits associated with motor function recovery. Longitudinal assessment of CMC amplitude and frequency variations could provide valuable insights into the dynamic process of motor system reorganization during recovery (48). Additionally, in the acute phase following stroke, motor evoked potentials (specifically event-related desynchronization, ERD) have demonstrated predictive value for assessing the potential of motor function recovery (49). This study aimed to identify EEG biomarkers of post-stroke brain function, with the strongest findings occurring in the high-frequency *β* band. The information potential of EEG, combined with its portability and maneuverability, may provide clinicians with an additional tool to facilitate the judgment of patient prognosis, the allocation of treatment and the evaluation of treatment effects. The current study adds to the body of evidence describing the clinical potential of EEG biomarkers in stroke rehabilitation and recovery.

From a practical perspective, EEG is easy to set up, offers excellent temporal resolution, and are widely available in clinical settings. Recovery after stroke is dependent on brain circuit reorganization and neuroplasticity (morpho-functional reorganization of the nervous system). Therefore, measures of brain dynamics associated with motor outcomes are valuable for characterizing patients, both as potential biomarkers and as components of multimodal measures that can inform mechanisms of recovery after stroke. Indeed, EEG measurements conducted after stroke can capture the reorganization of brain regions that facilitate clinical recovery by revealing dynamic changes in brain activity. EEG can be used in measures designed to combine changes in electrical cortical activity with a patient’s clinical deficits as assessed by a clinical rating scale, a potentially clinically significant approach. EEG measurements taken after a stroke can document the reorganization of brain regions that support clinical recovery, by revealing changes in interhemispheric balance, changes in activity in regions associated with damaged areas, and the reorganization (7) of body representation maps. Combined with data from clinical assessments, EEG based quantifiers can help maximize recovery potential by supporting prognostic accuracy through patient characteristics and facilitating the identification of rehabilitation strategies appropriate to the subject’s functional state.

To our knowledge, this is the first study to explore the dynamic tracking of resting EEG changes during routine rehabilitation intervention. EEG was measured at three time points: Day 1(T0), Day 7(T1) and Day 14 (T2). We compared behavioral scales—FMA-UL, ARAT, MBI, and NIHSS—in 113 patients before and after the routine rehabilitation intervention. The results demonstrated significant improvements in upper limb function, daily living ability, and neurological function following the intervention compared to baseline. The correlation between EEG parameters and behavioral assessments, including FMA-UL, ARAT, MBI and NIHSS were analyzed.

In terms of EEG results, we also compared the relationship between BSI and behavioral data. We found that BSI was negatively correlated with behavioral data in Brunnstrom-UL, FMA-UL, FMA HAND and MBI after intervention, indicating that with functional recovery, the BSI value showed a negative correlation. The asymmetrical differences in bilateral brain gradually narrowed, this is consistent with previous research (18). The changes in excitability and connectivity following a stroke, encompassing both the acute and chronic phases, have been well-documented. Previous studies indicate that variations in interhemispheric coupling strength within homologous regions are associated with the degree of motor function recovery, particularly between primary motor areas (50). This may explain the observed downward trend in BSI values. Under normal resting conditions, there is an active interaction network between the two hemispheres of the brain; however, alterations in cortical activity during resting states post-stroke are linked to motor dysfunction during movement (51). In this study, EEG results demonstrated a reduction in bilateral interhemispheric coherence after rehabilitation interventions. This suggests that plastic changes within the brain’s integrative functional network are related to upper limb recovery following a stroke (52). Collectively, all spectral findings suggest that relevant connectivity involved in restoration (or enhancement) positively impacts subsequent upper limb movement recovery. This presents new opportunities for future research into functional connectivity within brain networks following strokes.

The analysis between the two scales and the three different EEG frequency bands found that only the *β* band was positively correlated with behavioral data, while no significant correlations were detected in the other bands. In this study, the increase in beta activity appeared to be associated with favorable clinical outcomes, suggesting that higher frequency bands of brain activity may contribute to better patient responsiveness and receptivity, which may be associated with better patient responsiveness and receptivity (5). Beta activity in the 20–30 Hz band was associated with cortical output from pyramidal neurons, suggesting that beta band EEG measurements may serve as a potential biomarker of CST injury (53). The β band shows a positive correlation in the unaffected hemisphere, possibly reflecting compensatory mechanisms in stroke recovery in patients with subacute stroke, the brain activates neuroplasticity mechanisms to facilitate functional reorganization. The unaffected hemisphere compensates for the impaired functions of the damaged brain regions, thereby maintaining motor functions. During this process of functional reorganization, the corresponding neural networks in the unaffected hemisphere become more active and efficient, leading to an increase in the power spectral density of the beta frequency band in EEG signals.

The aim of this study was to demonstrate that simple EEG measures can predict motor rehabilitation outcomes in patients with subacute stroke. The BSI indices are straightforward to calculate and interpret, making them practical for clinical use. Similarly, the β bands are simple values and can therefore provide valuable information for clinical decision making. However, further complementary EEG measurements, such as resting state connectivity analysis, should provide a more comprehensive overview of brain state and better predict motor recovery.

The main limitation of this study is the short hospital stay of the patients, which resulted in brief intervals between EEG tests, a high frequency of testing, and a relatively small sample size during the study design phase. However, it is important to note that despite the limited sample size, the study encompassed a diverse dataset, including three EEG tests, pre- and post-training FMA, ARAT, MBI, and NIHSS assessments, yielding a substantial volume of data. Despite the small sample size, we think our results show interesting trends in subacute patients and remain attractive given the lack of research in this area of study, and the inconsistent results found in the literature. Our results show interesting trends in patients during routine rehabilitation interventions in the subacute phase that have not been studied before. The results of this study should be regarded as the first exploratory insight into EEG indicators in predicting motor rehabilitation outcomes in patients with subacute stroke.

## 6 Conclusion

The results of this study suggest that EEG indicators may serve as valuable tools for predicting motor prognosis, providing valuable information for clinical decision making. Such information is essential for tailoring individualized treatment plans for patients. Although the results of this study suggest that patients with significantly elevated *β*-band energy spectrum are more likely to experience motor improvements, further studies in larger samples are needed to validate the role of EEG in predicting motor recovery.

## Data Availability

The raw data supporting the conclusions of this article will be made available by the authors, without undue reservation.
